# An innovative team-based weightloss competition to reduce cardiovascular and diabetes risk among Māori and Pacific people: rationale and method for the study and its evaluation

**DOI:** 10.1186/s40795-017-0199-2

**Published:** 2017-10-13

**Authors:** Marewa Glover, Anette Kira, Geoff Kira, Hayden McRobbie, Bernhard H. Breier, Rozanne Kruger, Jane Stephen, Mafi Funaki-Tahifote

**Affiliations:** 1 School of Health Sciences, College of Health, Massey University, Albany Highway, Albany, Auckland, 0632 New Zealand; 2 Wanganui, Manawatū New Zealand; 3 The Dragon Institute for Innovation, Auckland, New Zealand; 4 School of Sport, Exercise and Nutrition, College of Health, Massey University, Private Bag 102904, North Shore, Auckland, 0745 New Zealand; 5 Pacific Heartbeat, Heart Foundation, PO Box 17-160, Greenlane, Auckland, 1546 New Zealand

**Keywords:** Obesity prevention, Weightloss competition, Diabetes, Cardiovascular disease, Indigenous

## Abstract

**Background:**

Obesity rates for New Zealand (NZ) Pacific and Māori (NZ indigenous people) are among the highest in the world. Long-term results of weight management programmes for adults have been modest but primarily focused on individuals. This paper describes the rationale and methodology for a trial of a culturally tailored team-based weightloss competition conducted online with community level support.

**Methods/Design:**

A quasi-experimental design was used to compare an intervention and control group. Three six-month competitions with seven teams of seven Māori or Pacific people (*N* = 147) were run. Eligible participants were: Māori or Pacific, 16 years of age and above, obese (BMI ≥30 kg/m^2^) and either at risk of or already diagnosed with type 2 diabetes (HbA1c >50 mmol/mol) or cardiovascular disease.

The intervention facilitated group use of an internet-based competition offering financial incentives, education and support. The primary outcome was percentage of individual weight lost at 12-months. Secondary outcomes were percentage reduced total cholesterol and glycated haemoglobin (HbA1c). Data collected at baseline, 6-months and 12-months included: height, body weight, blood lipids and HbA1c, eating and dieting habits, family support, food access, alcohol use, nutrition literacy, activity levels, perceptions of weight, stress and sleep, and, perceived contagion effect. Process evaluation tasks will inform acceptability.

**Discussion:**

An attractive, easy to understand weight change programme that effectively reduces disease risk among Māori and Pacific is desperately needed. Web-based delivered support and information to largely self-directed teams could also ease exponential rises in costs to the health system.

**Trial registration:**

Trial Id: ACTRN12617000871347.

## Background

Obesity is associated with a multitude of illnesses and disease including cardiovasular disease (CVD), type 2 diabetes mellitus (T2DM), gout, depression, and several cancers [1]. New Zealand (NZ) has very high prevalence of obesity. For people who are 20 years or older, NZ males have an obesity rate of 28% and NZ women are at 30% [2]. Against global rates, NZ resident Pacific have the highest rate of obesity globally at 66.9% and Māori, indigenous New Zealanders, at 47.1% rank among the top 15 populations [3].

Internationally, population focused obesity reduction strategies appear to have little effect. No countries have achieved a significant decrease in obesity in recent decades [4]. Weightloss interventions combining reduced caloric intake and increased physical activity have been a primary strategy [5]. Meta-analyses show the effectiveness of these approaches but translation into routine healthcare practice and difficulty maintaining changes has undermined longer term benefits [6].

Indigenous people in colonised countries such as, the USA, Canada, Australia and NZ have higher prevalence of obesity than their non-indigenous counterparts [4]. Internationally, there have been some interventions for indigenous people, for reducing T2DM prevalence [5, 7–12] or that aimed to reduce obesity, CVD and diabetes risk [13–15]. These interventions most commonly included exercise [10–14] and/or dietary education [5, 9, 13, 14]. Other interventions, that have shown promise aimed at addressing food availability, [15] community healthcare worker outreach [8] or taught mindfulness [7, 16].

Despite the disproportionately high prevalence of obesity for Māori and Pacific people in NZ, only a few interventions have been designed specifically for Māori and/or Pacific adults. One large prospective survey and two pilot studies specific to Māori include the Ngati and Healthy community programme that was effective in reducing insulin resistance, [17, 18] the Vanguard pilot study, which utilised a Māori community health personal trainer and achieved weightloss [19] and a pilot community programme that improved insulin sensitivity, and reduced weight and systolic blood pressure [20]. For NZ Pacific people, three church-based pilot interventions have been conducted that showed promising effectiveness in reducing diabetes for Pacific people [21–23].

A systematic review found that most weightloss interventions focus on the individual [24]. They argue that obesity interventions need to focus wider than the individual and include interpersonal, organizational, or community levels. One randomized controlled trial found that group-based incentives for weightloss were more effective than individual incentives [25]. Furthermore, one type of intervention that has gained popularity and indicates effectiveness in the USA is a large-scale team weightloss competition [26]. However, we have not been able to find any published research interventions that incorporate team competitions for weightloss for indigenous people.

Previous research has found that recruiting and retaining indigenous people in controlled trials can be difficult [27]. However, involving indigenous people in all aspects of the trial and basing the intervention on an indigenous framework can mitigate difficulties (ibid). This paper describes the rationale, the underlying Māori framework and method for an intervention trial, called WEHI, that aims to test if a culturally and community-based team competition could be effective in prompting and maintaining weightloss through improving healthy eating behaviours and increasing physical activity. The competition will be trialled with Māori and Pacific adults, with a BMI of 30 or higher, identified as having or being at risk of developing T2DM or CVD. The predictions are:

- participants who take part in a team based weightloss competition tracked publicly online will experience:
  - ◦ greater average decrease in weight and waist circumference than the control group
  - ◦ greater improvement in plasma HbA1c and plasma cholesterol and lipids levels than the control group.

## Theoretical framework

We used Te Whare Tapa Wha [28] (the four sided house) as the underlying Māori theoretical framework. Te Whare Tapa Wha is an ecological model used to convey a Māori holistic understanding of health. The four sides of the house are te taha tinana (the physical realm), te taha hinengaro (the mental realm), te taha whānau (the family/social realm) and te taha wairua (the spiritual realm). Te ao tūroa (the long-standing environment) is used in addition to place the house within the wider socio-historical-political environmental context. The four sides of the house and its environment are interdependent.

### Te taha tinana (the physical realm)

Factors that are physical include age, ethnicity, height and genetic predisposition/makeup. These are not modifiable as such, although age and metabolism changes over time. Illness and injury are recognised barriers to physical activity that may not be modifiable without specialised intervention [29].

Sleep quantity and quality, the physical effects of stress and activity levels are defined here as modifiable ‘physical’ factors. As are behaviours such as eating, alcohol consumption, smoking and medicinal or recreational drug use [30].

In the Te Ao Tūroa realm, te taha tinana is affected by nutritional quality.

### Te taha hinengaro (the mental realm)

Some beliefs that drive individual choice are deeply held, such as religious or cultural beliefs, and may not be open to modification. In contrast, cooking methods, nutrition, portion sizes and cooking skills are modifiable, as long as the cultural context, knowledge and attitudes about food are acknowledged. Therefore, messages that work primarily on the psychological realm trying to change knowledge, beliefs and manipulating emotion and self-perception may be able to create cognitive dissonance and thereby motivation to change. Mental health disorders, such as depression and the psychological effects of illnesses, belong in this realm.

### Te taha whānau (family and social factors)

Socio-economic status is a key social determinant of obesity and adverse health outcomes [30]. Although income has an inverted U-shaped relationship with body weight, with extreme poverty associated with being underweight, and weight gain with income at higher levels being offset by demand for an ideal body weight [31]. Nutrition and food security has been defined as “all people, at all times, have physical and economic access to sufficient, safe and nutritious food to meet their dietary needs and food preferences for an active and healthy life” [32]. Nutrition security is often an issue for Māori and NZ Pacific whānau (families) [33]. The political reality is that for the term of this trial, income level is not likely to change and as such is not classified as a modifiable factor.

Whānau norms in terms of beliefs about food, eating, cooking, serving and sharing food may impact ability to change behaviours. Duty, in relation to food, includes beliefs about manaakitanga (the value of showing respect, generosity and care for others) and koha (reciprocal gifts) for Māori and fatongia for Tongans. Fatongia refers to the obligation to provide and the obligation to eat what is provided. Past whānau and cultural norms around gardening, food storage, cooking, eating and sharing food could offer protective factors or where poor diet has become intergenerational, be supporting less healthy practices.

Household modes of transport can similarly encourage ambulatory activity or be inhibitive of movement, but changing where people live in relation to their work or food sources and how they move between their necessary environments is not seen as modifiable for the purpose of this research. Whether people live in a city, close to food sources, or rurally is not modifiable.

At a Te Ao Tūroa level, supermarkets and grocery stores, and takeaway food outlets have been increasing in number and sales volumes year on year (2010–2013) while the percentage of smaller fresh fruit, vegetable, meat and fish stores have been reducing (2010–2012) [34]. Whilst supermarkets have a full range of foods to choose from, they and fast food restaurants are highly engineered spaces designed to manipulate product choice [35] and extra non-planned purchases [36]. Modifying this global phenomenon within this short-term pilot to redirect participants’ food choices back to ‘fresh’ ‘real’ food favouring vegetables is outside the scope of this intervention. We can, however, try to raise awareness of the marketing strategies shoppers are exposed to.

### Te taha wairua (the spiritual realm)

Matauranga Māori (Māori beliefs) that included cosmology of food and protocols to protect food sources have been diminished by the process of colonisation [33]. Most Māori have been cut-off from natural free food sources, and the knowledge about how to grow and gather and sustain food sources (ibid). Many food sources have been lost, for example, access to seabeds has been removed or regulated, birds that were once a food stock are endangered and protected, fish sources have been depleted by commercial fisheries or the larger population of NZ disrespecting quotas for fish and seafood. Traditional foods have been replaced with ‘modern delicacies’ such as canned ham. There has been a loss of tikanga (protocols) around food and a loss of the meaning of food [37]. The importance of gardening and feeding the people has been reframed so that providing people with commercially produced foods, has become as or more acceptable.

In conclusion, for this pilot, knowledge and behaviours need to change sufficiently within a 6 month period to result in lowered HbA1c and improvement in blood lipids. Thus, WEHI will focus on the identified most readily modifiable factors discussed: increasing physical activity, reducing excessive food consumption, reducing consumption of added sugar, increasing consumption of vegetables, increasing use of healthier cooking methods, improving knowledge of nutrition and ideal food portion sizes, increasing mindfulness and awareness of influences on dietary choices.

## Method and design

This was a quasi-experimental pilot study that compared an intervention group with a consecutive but separately recruited group of matched (on ethnicity, gender & age) control participants. A quasi-experimental design was chosen because of the funding amount available, and the purpose of the fund was to explore and test concepts. The study started in June 2016.

### Ethics approval and consent to participate

This study was conducted according to the guidelines laid down in the Declaration of Helsinki and all procedures involving human subjects/patients were approved by the Northern B Health and Disability Ethics Committee (16/NTB/101). Written informed consent was obtained from all participants.

### Participants and sample size

One of the objectives was to trial the intervention across a range of contexts to increase generalizability to a wider population. Therefore, three distinctly different regions were selected: an urban Māori population (Manawatū), a small town/rural Māori population (Northland), and a Pacific Island community from a major NZ city (Auckland).

In a previous trial of a team competition to support smoking cessation among Māori and Pacific, ten members were required per team [38]. One learning from that trial was that it was difficult to recruit the required number to make a team and the teams, once formed, had difficulty organizing team meeting times to suit 10 people [39]. Therefore, for the WEHI trail, it was decided that seven people per team may be a more manageable number. The funder had requested that 150 people participate in the competition, hence seven teams of seven, for each region, totalling 147 participants were to be recruited.

A similar number (*N* = 150) of people were to be recruited as control participants.

### Eligibility and exclusion criteria

The eligibility criteria were Māori or Pacific people, aged 16 years of age and above, who were obese (defined as having a body mass index (BMI) ≥ 30 kg/m^2^), and were either: 1) at risk of developing T2DM (glycated haemoglobin (HbA1c) = 41–49 mmol/mol) or CVD (defined as having an ‘elevated cholesterol’, compared to the laboratory's normal range, in a blood test done during the last 12 months), or, 2) who had been diagnosed with T2DM (HbA1c >50 mmol/mol) or CVD by a health professional. Potential participants had to be willing to see their doctor for a blood test for HbA1c and cholesterol. Additionally, enrolment in the WEHI trial required each team member to commit to participation in the intervention phase of 6 months duration, and the follow-up study phase of a further 6 months.

People were excluded if they were younger than 16 years of age, pregnant or planning a pregnancy within the next year, or women who had a live baby in the past 6-months and who were breastfeeding. People who had type 1 diabetes or who self-reported currently smoking tobacco or marijuana, or who used nicotine replacement products or nicotine from any other source (e.g. electronic devices), were excluded. Nicotine is a known appetite suppressant and could confound the results [40].

### Recruitment

#### Trial regions

The geographical regions for this study were chosen for their:

- Presence of a large proportion of Māori and/or Pacific peoples;
- Availability of support services (behavioural change and weight management service providers); and
- Regional level interest from community groups to assist with recruitment and intervention delivery.

Local regional health providers were contracted and trained to co-ordinate promotion of the programme, recruitment, screening and data collection. They appointed a regional co-ordinator (RC) who supported the teams with information on how the competition works, how to use the website and how to access support from local services to achieve behavioural changes.

#### Recruitment procedure

Presentations to and notices seeking participants were distributed through community organisations and health providers using the contracted provider’s existing networks. We allowed eight weeks for recruitment. Each region was able to choose its own competition start date. All teams within a competition region started on the same day. Recruitment of control participants occurred concurrently. However, because we anticipated that recruiting controls would be more difficult than recruiting intervention participants, control recruitment was done over an extended time period (four months).

#### Control participants

Convenience sampling was used initially to find control participants. People who responded to recruitment activities promoting the study were initially screened for entry into the competition arm of the study. If interested people could not or did not want to take part in the team competition, they were asked to be involved in the study as part of a comparison group (the controls). Additional promotion was carried out by the RC’s to find sufficient numbers of controls. A sampling frame was built based upon the demographic profile of intervention participants and recruitment of controls was checked against this in an iterative way to guide ongoing recruitment of a control group matched to the intervention group.

Control participants were offered one entry into a prize draw (1 per region) for seven vouchers of $50 for completing the baseline, six-month follow-up questionnaire and the 12-month follow-up.

### Intervention

The intervention was based on a successful team smoking cessation competition - WERO, [38] which included the following components: group support, financial incentives, competition and internet delivered education and support. How the components were used for WEHI is covered in more detail below.

In addition to the components included in WERO, we provided individual WEHI participants with a diary to facilitate tracking of daily challenge activities. Teams were supported with team activity summary sheets and a fridge magnet as a mnemonic to remind participants of the daily challenges. The teams were supplied with a set of bathroom scales to use as they wished to track their weightloss progress.

WEHI participants were encouraged and supported to use whatever non-surgical methods, diets, programmes or health and fitness providers they wanted and could access at their own cost. No particular dietary intervention was promoted, although Ministry of Health guidelines on healthy eating and physical activity were followed for the development of tips and team daily and weekly challenge activities. Furthermore, the use of supplements were discouraged as many products are costly with no known scientific basis of efficacy. Radical short-term approaches like extended periods of fasting, or ‘fad’ diets raising health concerns in the obesity sector were also discouraged. WEHI was focused on helping people make changes they could sustain.

#### Group support

WEHI participants competed in teams of seven, which boosted peer social support [38] and enabled the dynamics of whānaungatanga (a relationship through familial and shared experiences) to operate. Each team were self-directing in terms of organising their team meetings and activities. Teams received support from the RC though this needed to be equitably provided to each of the teams to avoid advantaging one team over the others.

#### Incentives

In each region, three (NZ$) prizes were offered:

- Greatest progress at 2 months - $1000
- Greatest progress at 4 months - $1000
- Greatest progress at 6 months - $3000

If more than one team drew, then they split the prize.

The prizes were paid to the team’s nominated charity or community organisation. This fundraising aspect was expected to appeal to participants’ existing sense of duty to raise funds for cultural or local community events and other needs. This was expected to provide additional motivation over and above any motivation to lose weight for personal benefit.

Donated products, such as magazines and herbal tea samples, were sought from companies and provided to teams as further incentives.

#### Competition

Team progress at each follow-up was calculated as follows:

1. Number of team members who had lost ≥4 kg weight in the preceding 2 months
  PLUS
2. Number of team members who had lost ≥3 cm in waist circumference during the preceding 2 months
  PLUS
3. The team’s position on the competition scoreboard based upon the teams’ participation and completion of daily and weekly challenges for each 2-month assessment period (lowest ranked position = 1 to highest ranked position = 7).

#### Internet delivered education and support

**Fig. 1:**
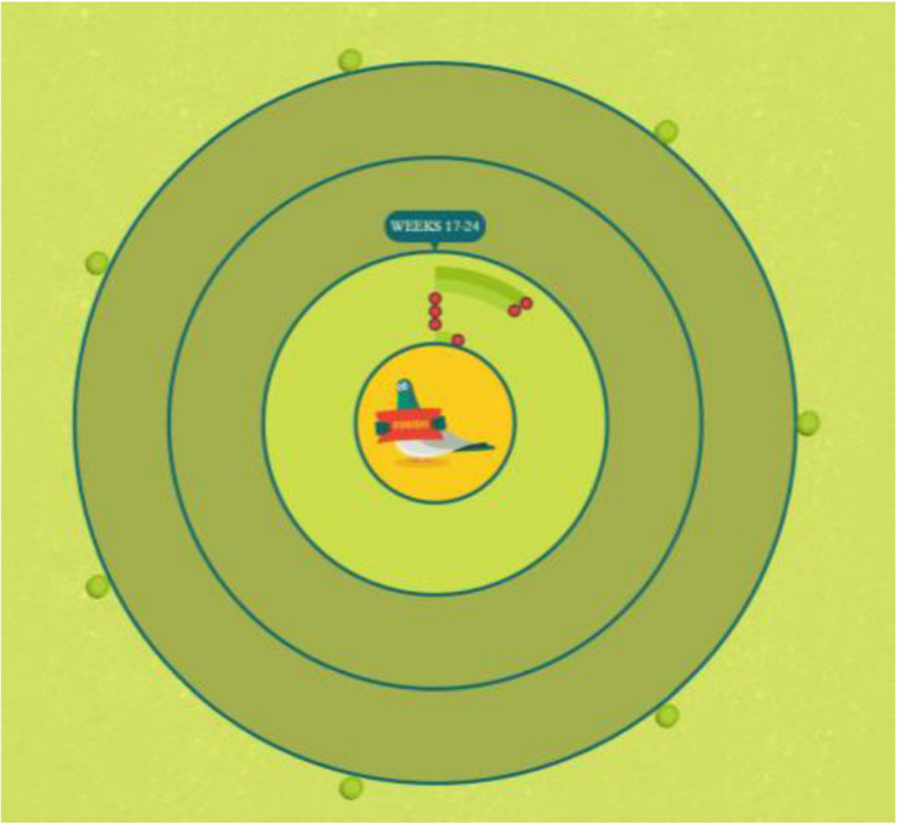
The wehi scoreboard showing the progress of the different teams in a region

The website displayed the progress of each team, as a whole, on the competition scoreboard. The scoreboard was based on a traditional Māori team ball game called Ki-o-Rahi which is similar to a British game called Bulldog, or bullrush. The team scoreboard was publicly available on the WEHI website (Fig. 1). Being a member of a team and having the team’s progress displayed on a public website was thought to have an element of ‘pledging’ to it. The progress of individuals was not displayed on the website.

Each team also had a dedicated team page, which was publically visible, where they were encouraged to post questions for the researchers, comments and photos about their team activities and support for each other. Supporters could also comment on team posts.

The website provided new information daily in the style of tips on how to lose weight, how to stay motivated, how to choose between foods and drinks, and how to increase the amount of physical activity in their day. Questions posed by participants could be answered on the team’s page, but could also be fashioned into a 'Tip of the Day'. Participant’s questions or comments about difficulties and barriers to change and trigger situations on their team page could also be answered and commented on by the study team.

The intervention group were provided with information and motivation to:

- Improve dietary habits, for example their awareness of portion sizes, healthier food choices and healthier cooking methods.
- Increase physical activity levels.
- Make other behavioural changes such as reducing alcohol consumption, reducing stress and getting sufficient and better quality sleep.

### Measures

#### Outcome measures

The primary outcome was percentage of individual weight lost.

Secondary outcomes were:

- greater reduced risk for CVD and T2D for the intervention versus control group as indicated by reduced HbA1c and improvement in lipid levels.
- The intervention group will, on average, experience a greater reduction in BMI.

#### Anthropometry

Basic anthropometric measurements (height, weight and waist circumference) of all participants was overseen by the RA, who was trained. The equipment used for measurements were SECA813 digital floor scale, [41] SECA portable stadiometer Height Rod [42] and SECA ergonomic girth measuring tape [41]. Based on those measures BMI was calculated as (weight (kg) ÷ (height (m) x height (m)) at baseline, 6 months, 12 months.

For weighing, the participants needed to be wearing light clothing and no shoes.

#### Blood test

All participants were required to visit their general practitioner (GP) at baseline, 6- and 12- month follow-ups to have blood tests measurement of HbA1c, total cholesterol, HDL cholesterol, LDL cholesterol and triglycerides. We gave participants a letter to take to their doctor which explained the study and what it involved. The letter explained the need for the blood test information and asked the doctor to share that with us. The letter incorporated a patient consent form, to be signed by the participant, giving permission for the doctor to share the blood test results with us (and how this could happen). If participants had recently (in the previous 2 weeks) had the required blood tests completed, we asked them to have their doctor share those results with us instead of repeating the tests.

#### Questionnaire

The screening questionnaire was administered by the RC. The questionnaires contained questions on: eating habits, dieting/weightloss methods used, family/whānau cooking & social support, food access, alcohol use, nutrition literacy, activity levels, perceptions of weight, stress and sleep and perceived contagion effect. RCs were trained in data collection and were available to assist with questionnaire completion for those who found it difficult to complete. RC’s were also to check that all data fields had been completed correctly.

Survey items from existing research were reviewed and selected for inclusion using the following criteria:

- relevance for the aims of this study,
- potential to compare with existing relevant literature,
- reliability,
- brevity and comprehension for a lay public with low health literacy.

Some previously developed survey items were adapted, reworded or expanded to increase relevance to the NZ Māori and Pacific participant population. For example, pinto beans are not a staple food among Māori and Pacific peoples in NZ. Beans, such as those used in baked beans, were substituted.

The questionnaires were piloted on three Māori and three Pacific people of different age groups to test item comprehension before use. Some additional instructions were added and formatting changed to ease comprehension.

#### Acceptability interviews

Some process evaluation questions will be asked at the 6-month follow up with a sub-sample of intervention participants and key stakeholders to assess acceptability and assist formulation of recommendations for further research, policy or programme planning.

### Data collection points

In addition to the outcome measurements, participants in the intervention group were required to provide their weight and waist measurements at two and four months into the competition. Along with the daily challenge activity data, these results were used to calculate the winner of the 'greatest progress at' 2, 4 and 6 months.

Participants in the intervention group was followed up after 6 months i.e. at the end of the competition. Control group participants were followed up six and twelve months from the date of their baseline interview.

The various points at which data was collected, is shown in Table 1.

**Table 1:** Data collected at each collection point (I = intervention group, C = control group)

	Screening	Baseline	2 & 4 Months	6 Months	12 Months
Height	I, C				
Weight	I, C		I	I, C	I, C
Pregnancy status/intention^b^	I, C				
Smoking/nicotine use	I, C				
Diagnosis questionnaire	I, C				
Demographics^a^		I, C			
Biophysical measures					
Plasma HbA1c		I, C		I, C	I, C
Plasma Cholesterol & lipids		I, C		I, C	I, C
Waist Circumference		I, C	I	I, C	I, C
Questionnaire measures					
Eating habits		I, C		I, C	I, C
Dieting/weight loss methods		I, C		I, C	I, C
Family/Whānau Cooking & Social Support		I, C		I, C	I, C
Food access		I, C		I, C	I, C
Alcohol use		I, C		I, C	I, C
Nutrition literacy		I, C		I, C	I, C
Activity levels		I, C		I, C	I, C
Perceptions of weight		I, C		I, C	I, C
Stress and sleep		I, C		I, C	I, C
Perceived contagion effect					I
Acceptability interviews					
Acceptability of intervention					I
Key stakeholder perceptions					I

^a^ethnicity, age, gender, SES, town of residence, household composition

^b^only for women of child-bearing age

### Data management

Descriptive statistics will be produced for the intervention and control groups: average age overall, average age by competition region and by ethnicity; proportion of males overall and by competition and by ethnicity prevalence of self-reported T2DM and CVD; BMI: distribution by highest educational level obtained and by socioeconomic status.

Baseline participant characteristics for the intervention and control groups will be compared for differences using chi-squared tests (categorical variables) and ANOVA (continuous variables).

BMI will be analysed according to standard World Health Organisation categories: obesity I (30–34.9), obesity II (35–39.9), and obesity III (≥40).

Inductive thematic analysis will be used to identify major themes in qualitative data.

## Discussion

This paper described how a weightloss competition has been designed to be suitable for Māori and NZ Pacific people. Competition and incentives have shown potential in encouraging weightloss, however, no previous study has investigated if a weight-loss competition that is culturally salient and pragmatic for Māori and NZ Pacific people would be effective in reducing CVD and T2DM risk and prevalence. The present intervention differs from previous team weightloss competitions in that it is based on an indigenous framework, which was effective in encouraging smoking cessation [38]. Intervention design and content needs to support Māori and Pacific people’s aspirations to achieve improved health and well-being, which includes protection of cultural knowledge, practices and language [43].

Māori and Pacific health providers have significant understanding of their populations and the context impacting their health. WEHI trials an intervention framework that generates new opportunities for the integration of local, cultural and tribal tailoring of content as the resources available, for example gyms and swimming pools, which would assist in supporting the “environment realm” of Te Whare Tapa Wha, vary per region.

In addition to being designed in a culturally appropriate manner, interventions also need to align with current Government strategies and be able to be delivered within existing health delivery structures. The WEHI intervention aligns with the new NZ Health Strategy, in terms of supporting NZers to become health smart, which includes enabling individuals to access health information and care via internet and mobile technologies [44].

The questionnaires, pattern of daily challenges completed over time and information collected from competition teams in response to weekly challenges, will provide insight into the barriers to weightloss and support participants' need, not only for WEHI, but also for making other lifestyle changes that influence obesity. Furthermore, testing the competition in rural and urban settings will increase generalisation to different settings. Interest in the use of new internet based interventions is high for the promised efficiencies, but use of effective interactive websites can only deliver cost efficiencies if used as required. It remains to be seen if rurally based and/or low IT users access this component.

A major strength of this intervention is that it is culturally salient and pragmatic. It is based on a concept that has been proven successful in smoking cessation. Furthermore, it involves health workers in the intervention, meaning that they are experienced with the intervention, should it prove successful and is adopted for wider implementation.

There are also limitations. The project term of 18 months was short for a trial requiring culturally appropriate relationship building processes to be followed sufficiently [27]. If insufficient presentations explaining the research and intervention are done in the target communities, recruitment could be undermined (ibid).

There is a dearth of literature specific to Māori and Pacific on how to reduce obesity and risk of CVD and T2DM. Our study will provide much needed information on the topic and on how to design interventions and future trials for obesity prevention and/or treatment.

## Abbreviations

BMI: Body mass index
CVD: Cardiovascular disease
GP: General practitioner
NZ: New Zealand
RC: Regional co-ordinator
T2DM: Type 2 diabetes mellitus
